# Sonochemical synthesis of cellulose/hydroxyapatite nanocomposites and their application in protein adsorption

**DOI:** 10.1038/s41598-018-25566-7

**Published:** 2018-05-29

**Authors:** Lian-Hua Fu, Chao Qi, Yan-Jun Liu, Wen-Tao Cao, Ming-Guo Ma

**Affiliations:** 1 Beijing Key Laboratory of Lignocellulosic Chemistry, College of Materials Science and Technology, Beijing Forestry University, Beijing, 100083 PR China; 2 Guangdong Key Laboratory for Biomedical Measurements and Ultrasound Imaging, Laboratory of Evolutionary Theranostics, School of Biomedical Engineering, Health Science Center, Shenzhen University, Shenzhen, 518060 China; 3 Key Laboratory of Optoelectronic Devices and Systems of Ministry of Education and Guangdong Province, College of Optoelectronic Engineering, Shenzhen University, Shenzhen, 518060 China

## Abstract

Hydroxyapatite (HA) is the main mineral constituent in the hard tissue of vertebrate, which is recognized as an important biomedical material owing to its excellent bioactivity and biocompatibility. Herein, we report a facile and green sonochemical route for the rapid synthesis of cellulose/HA nanocomposites in NaOH/urea aqueous solution. The *in vitro* behavior of the cellulose/HA nanocomposites was studied to evaluate the biological response of the nanocomposites following immersion in simulated body fluid for various periods (maximum of 28 days). The HA crystals formed on the surface of the nanocomposites were carbonate-containing apatite, which is similar to the naturally occurring calcium phosphate materials. The HA nanosheets (assembly of nanorods) were mineralized on the surface of the nanocomposites, and maximum mass of the nanocomposites was reached 1.82 times of initial mass after 28 days of soaking. Moreover, the as-prepared cellulose/HA nanocomposites have good cytocompatibility, and show a relatively high protein adsorption ability using hemoglobin as a model protein. These results indicate that the as-prepared cellulose/HA nanocomposites are promising for applications in various biomedical fields such as tissue engineering and protein/drug delivery.

## Introduction

Hydroxyapatite (HA) is the main mineral component in the hard tissue of vertebrate bones and teeth as well as the most stable calcium phosphate phase under physiological conditions^1,2^. For decades, synthetic HA has received great research interest owing to its excellent biocompatibility, bioactivity, and osteoconductivity^3–5^. These excellent properties bring it promosing applications in bone repair^6^, tissue engineering^7,8^, drug/protein/gene delivery^9,10^, and other biomedical fields^11–13^. However, the poor mechanical properties (i.e. low flexural strength and fracture toughness) of pure HA limited its applications^4,5^. Inspired by natural bone, which was considered as a fiber reinforced hybrid material composed of collagen fibers and HA minerals^3,5^, many polymer matrices were introduced to fabricate HA-polymeric composite materials to improve the mechanical properties and biological properties of these materials, such as chitosan^14^, agarose^15^, collagen^16^, polyesters^17,18^, cellulose and its derivatives^19–21^. Among them, cellulose has received more and more attention due to its good mechanical strength as well as excellent biocompatibility, chemical stability, nontoxicity, low-cost, and easy fabrication into various morphologies with adjustable interconnecting porosity^22,23^. Moreover, the HA nanostructures with high specific surface area and unsaturated atoms can effectively interact with cellulose, resulting in the enhanced properties of their nanocomposites^24^.

Sonochemical method has been recognized as a promising strategy for the preparation of materials with novel morphologies and properties, owing to its features of intense local heating, high pressures, and extremely rapid cooling rates^25,26^. For instance, the vesicle-like nanospheres of amorphous calcium phosphate^27^, CdSe hollow spherical assemblies^28^, HA nanoflowers^29^, Fe_3_O_4_/SiO_2_ core-shell nanoparticles^30^, mesoporous spheres of calcium silicate hydrate^31^, and hollow structured zinc phosphate nanoparticles^32^ were obtained by sonochemical method. Recently, the sonochemical method has been developed in the preparation of cellulose-based nanocomposites, such as cellulose/CaCO_3_, cellulose/Mn_3_O_4_, and cellulose/Cu(OH)_2_/CuO hybrids^33–35^. It was found that sonochemical method do more favors to the synthesis of CaCO_3_ crystals with pure phase, uniform size and morphology, compared with the microwave-assisted or oil-heating method^26,33^. Moreover, ultrasonic irradition can enhance amorphization during synthesis of calcium phosphate material^36^, and small nanoparticles with narrow size distribution can be achieved by introducing ultrasonication into batch-carbonation reaction^37^. However, to the best of our knowledge, the production of cellulose/HA nanocomposites by sonochemical method for biomedical applications has not been reported.

**Figure 1:**
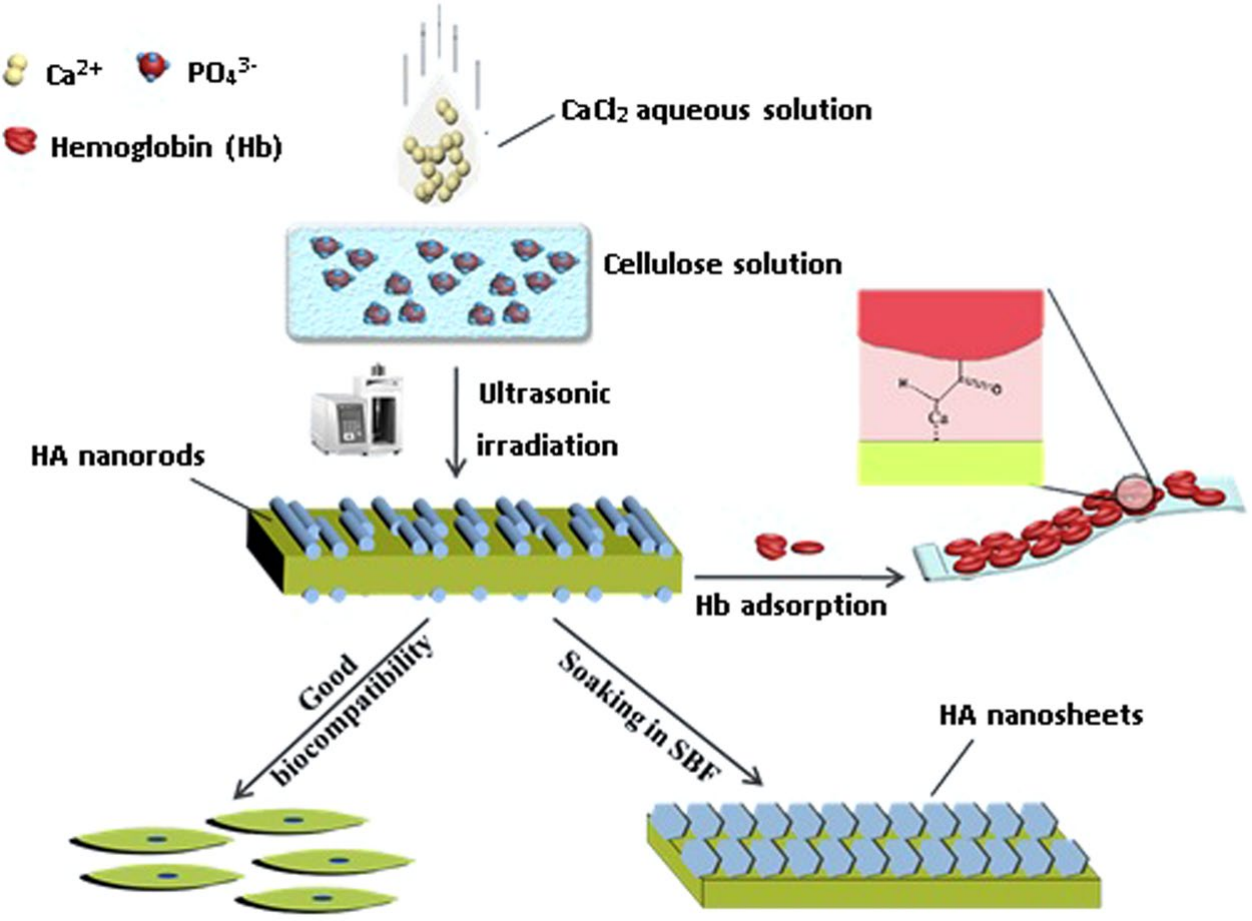
Schematic illustration for the preparation of cellulose/HA nanocomposites by the sonochemical method.

In this study, the cellulose/HA nanocomposites were synthesized by a facile and green sonochemical route in NaOH-urea aqueous solution (Fig. 1), which possessed at least three advantages: (i) the sonochemical method can drive chemical reactions due to the acoustic cavitation, and it is a greener and promising method compared with conventional methods such as oil heating; (ii) in comparison with other polymer matrices such as collagen, cellulose can easily be obtained from plants, which is an abundant and renewable biopolymer in the biosphere; (iii) cellulose could not only serve as the substrates for HA crystals, the inter- and intramolecular hydrogen bonds network of cellulose might serve as the structure directing agent that control the growth of HA crystals. The HA crystals are found bound to cellulose substrates, and the interaction between cellulose OH groups and HA can effectively enhance the mechanical properties of the nanocomposites, and stabilize it. The *in vitro* behavior of the as-prepared cellulose/HA nanocomposites was studied to evaluate the biological response of the nanocomposites following immersion in simulated body fluid (SBF) for various periods (maximum of 28 days), and the nanocomposites exhibit high ability to induce the formation of apatite. Moreover, the cytotoxicity tests demonstrate that the cellulose/HA nanocomposites have a high biocompatibility. The as-prepared cellulose/HA nanocomposites was explored for potential application for protein adsorption using hemoglobin (Hb) as a model protein, which showed a relatively high Hb adsorption ability. Thus, the as-prepared cellulose/HA nanocomposites are promising for various biomedical applications such as tissue engineering and protein/drug delivery.

## Results and Discussion

### Characterization of the cellulose/HA nanocomposites

**Figure 2:**
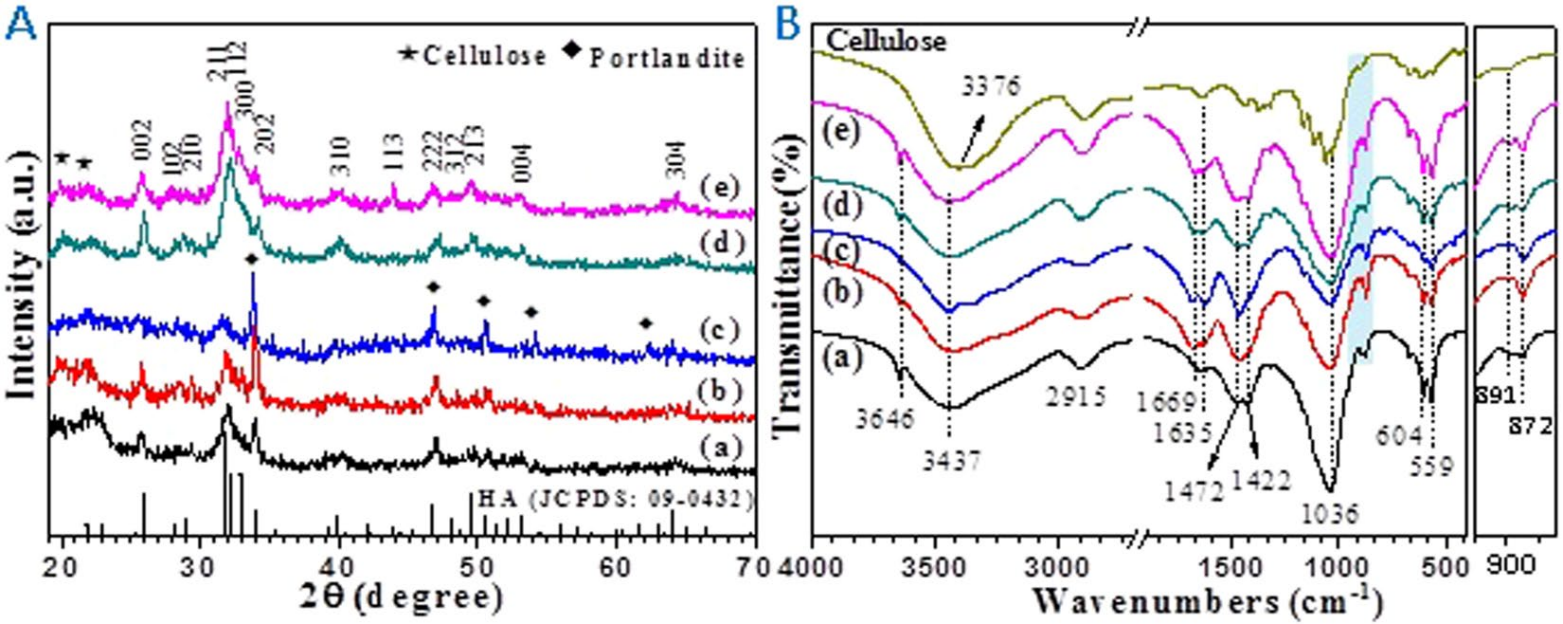
(**A**) XRD patterns and (**B**) FT-IR spectra of the as-prepared cellulose/HA nanocomposites: (a) M10; (b) M30; (c) M40; (d) M50; (e) M70.

The crystal phases of the as-prepared cellulose/HA nanocomposites are characterized by XRD, as shown in Fig. 2A. All of the samples exhibited diffraction peaks around at 2*θ* = 20.05° and 21.90°, which are belonged to cellulose (JCPDS 03-0226), indicating that the microcrystalline cellulose was irreversibly converted to cellulose II after treated with NaOH/urea solvent^38^. From Fig. 2A, the products prepared with HA/cellulose mass ratio of 10%, 30%, and 40% are consisted of a mixture of HA (Ca_10_(PO_4_)_6_(OH)_2_, JCPDS 09-0432) and portlandite (Ca(OH)_2_, JCPDS 04-0733). When increasing the HA/cellulose mass ratio to 50%, all the diffraction peaks can be indexed to the well-crystallized HA (Fig. 2Ad). Further increase the HA/cellulose mass ratio to 70%, the HA with higher crystallinity obtained, as more diffraction peaks of HA occurred (Fig. 2Ae).

The Ca/P molar ratios are 1.78, 1.89, 1.89, 1.87, and 1.90 corresponding to samples M10, M30, M40, M50, and M70, respectively (Table 1). All of the values are higher than the settled Ca/P molar ratio of 1.67 in the experimental section, this result can be explained by the influence of the byproduct portlandite in sample M10, M30, and M40, since the Ca and P contents analyzed by ICP represented the total element contents in the minerals. On the other hand, [PO_4_^3−^] in HA may be partly replaced by [CO_3_^2−^] from urea, resulting in the higher Ca/P molar ratios. Since the sonochemical method is based on the acoustic cavitation, which involves the formation and implosive collapse of bubbles in a solution, leading to intense local heating, high pressure, and extremely rapid cooling rate (10^10^ K s^−14^)^25,26,33^. It was reported that the temperature of the inside collapsing bubbles and interfacial region was measured to be about 5000 K and 1900 K, respectively^39^. Undoubtedly, the temperature of the reaction system will be continuously rising during the reaction. It’s also the reason why the reaction system was cooled in an ice bath during the ultrasonic irradiation and the sonication was opened 2 s and closed 2 s for protecting the instrument. As a diamide of carbonic acid, urea is thermally instable, and can be decomposed into melamine, ammonia, and carbon dioxide during the ultrasonic irradiation. The gaseous product of carbon dioxide can be converted into carbonic acid, and then ionized into CO_3_^2−^ ions, under the strong alkaline condition^24^.

**Table 1:** The experimental parameters for the preparation of typical samples, and the components of mineral and Ca/P molar ratios in the as-prepared nanocomposites.

Sample No.	CaCl_2_ [g]	NaH_2_PO_4_·2H_2_O [g]	Mass ratio of HA/cellulose	The phase of mineral	Ca/P molar ratio
M10	0.111	0.093	10%	HA, portlandite	1.78
M30	0.332	0.280	30%	HA, portlandite	1.89
M40	0.442	0.373	40%	HA, portlandite	1.89
M50	0.553	0.466	50%	HA	1.87
M70	0.774	0.653	70%	HA	1.90

It should be noted that the byproduct portlandite exists in samples M10, M30, and M40, while disappeared in the products prepared with higher HA/cellulose mass ratios of 50% (sample M50) and 70% (sample M70). It is well known that the solubility product (Ksp) of HA is far less than portlandite under the same temperature. Generally, the HA should be formed preferentially in the reaction system (containing Ca^2+^ and PO_4_^3−^ ions under alkaline condition) and then the portlandite. Cai *et al*. proposed that the cellulose dissolved at −12 °C could induce a fast dynamic self-assembly process among solvent small molecules (NaOH, urea, and water) and the cellulose macromolecules^40^. From the optical photographs, it is obviously that the NaOH/urea aqueous solution system increased in viscosity after dissolving cellulose (Fig. S1). Thus, at the beginning of reaction, Ca^2+^ and PO_4_^3−^ ions were difficult to diffusion in the reaction system owing to the high viscosity of the cellulose solution. As a result, the Ca^2+^ ions can be grasped by OH^−^ ions in the solution, and portlandite was obtained (Fig. 2Aa–e). With the elevation of temperature during the reaction producer, the viscosity of the solution rapidly decreased and induced the synthesis of HA. What’s more, it is reported that portlandite can transform into HA under certain conditions in the presence of PO_4_^3−^ ions^41,42^. Therefore, portlandite could be transformed into HA when surrounded by a great number of PO_4_^3−^ ions. This can be responsible for the samples prepared using high HA/cellulose mass ratios of 50% and 70% (Fig. 2Ad and e), where the mineral was HA without any impurity.

The FT-IR spectra of pure cellulose and the as-prepared cellulose/HA nanocomposites are shown in Fig. 2B. From which, all of the samples prepared with HA/cellulose mass ratios of 10–70% exhibited similar adsorption peaks. The characteristic bands of cellulose are observed around at 3437 cm^−1^ (stretching vibration of OH), 2915 cm^−1^ (stretching vibration of C–H groups), 1635 cm^−1^ (bending mode of absorbed moisture), 1375 cm^−1^ (O–H bending), 1164 cm^−1^ (C–O antisymmetric bridge stretching), and 891 cm^−1^ (*β*–glycosidic linkages between glucose units), which are consistent with that of pure cellulose. While the absorption peaks at 1110, 1056, and 1034 cm^−1^ of cellulose are overlapped with the peaks of HA (the *v*_3_ vibration of O–P). A small peak at 1669 cm^−1^ related to carbonyl stretching occurred in the cellulose/HA nanocomposites, which was resulted from the hydrolysis and peeling reaction of cellulose under strong alkaline conditions (NaOH/urea aqueous solution)^43^. Otherwise, the peaks located at 604 and 559 cm^−1^ are ascribed to the *v*_4_ bending mode of O–P–O in HA, and the peak at 3647 cm^−1^ is attributable to the surface P–OH groups^4,29,44^. The peaks appeared at 1472 (*v*_3-3_ CO_3_^2−^), 1422 (*v*_3-4_ CO_3_^2-^), and 872 cm^−1^ (*v*_2_ CO_3_^2−^) in all of the samples, suggesting that the [PO_4_^3−^] was partly replaced by [CO_3_^2−^] from the urea, which is consistent with our previous reports^22,24^. Moreover, the appearance of these peaks just confirmed the speculation about the high Ca/P molar ratios aforementioned.

It should be noted that the spectra from 3700 to 3100 cm^−1^ in the nanocomposites hindering the observance of the stretching vibration of cellulose OH groups at 3376 cm^−1^. The effects of HA on the cellulose OH groups are investigated by spectral deconvolution, and the results are shown in Fig. S2. From which, one can see that the peaks of cellulose OH groups shifted to lower wavenumbers in the presence of HA. The OH absorption peak at 3361 cm^−1^ shifted to 3249 cm^−1^, and the peak at 3228 cm^−1^ shifted to 3123 cm^−1^. This blue shift of cellulose OH groups involved with intramolecular hydrogen bonding, indicating that the cellulose OH groups are bonding with HA^44^. The formation of chemical bond between cellulose and HA can stabilize the nanocomposites, which was a crucial property for applications in gene/drug/protein delivery, and bone tissue engineering^44^.

**Figure 3:**
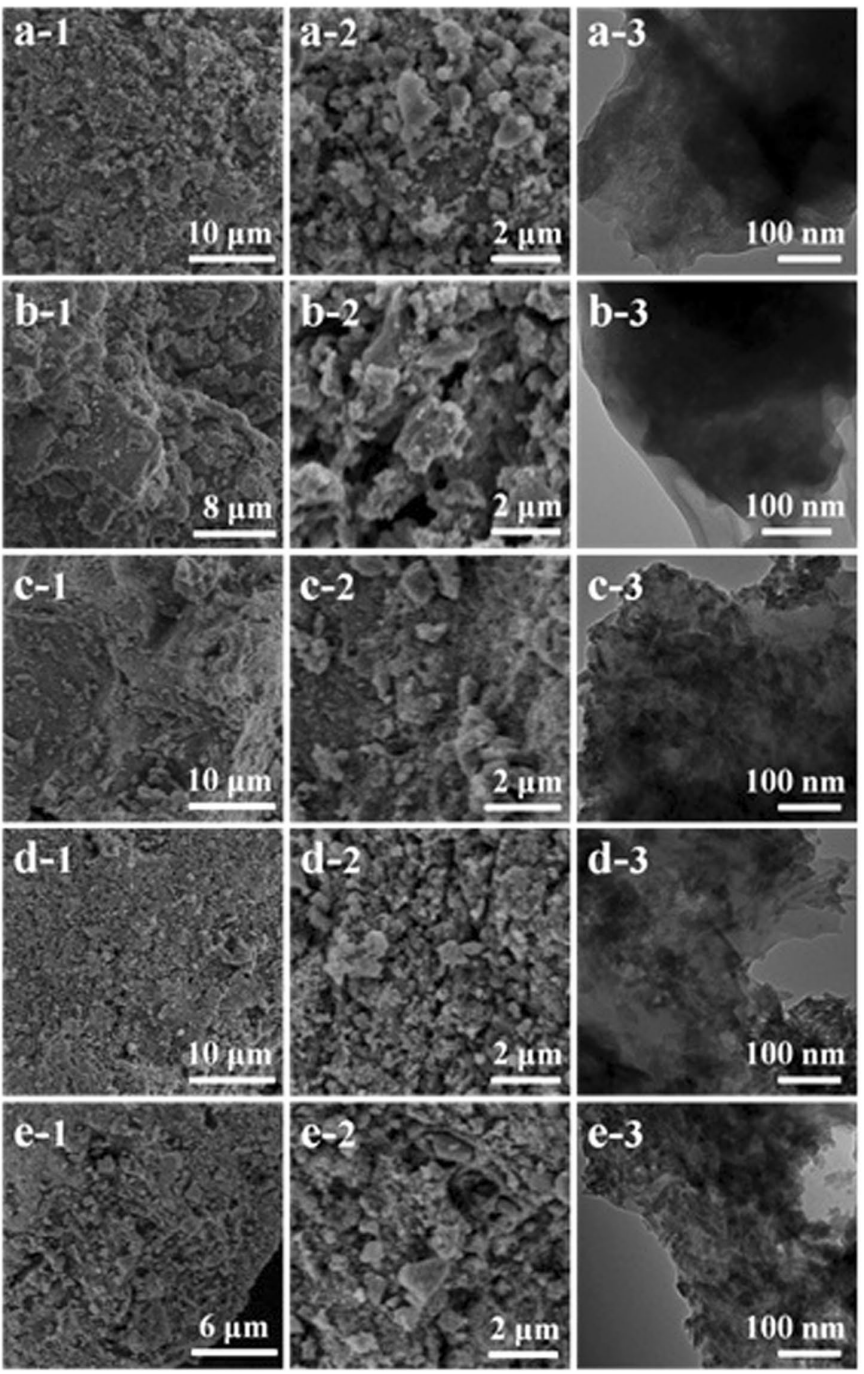
FE-SEM (left and middle columns) and TEM (right column) images of the as-prepared cellulose/HA nanocomposites: (**a**) M10; (**b**) M30; (**c**) M40; (**d**) M50; (**e**) M70.

The morphology of cellulose/HA nanocomposites was observed by FE-SEM and TEM, as shown in Fig. 3. The cellulose used in this study has a relatively dense structure with smooth surface (Fig. S3), while after the ultrasonic treatment, cellulose lost its original morphology and the surface became coarse (Fig. 3a–e, the FE-SEM images). Cellulose substrates in all of the samples were covered by a great number of mineral particles in uneven sizes, which was a mixture of HA and portlandite for samples M10, M30 and M40, and a pure phase HA for samples M50 and M70, as investigated by XRD patterns (Fig. 2A). From the high magnification FE-SEM images, one can find that when increasing HA/cellulose mass ratio to 50% and 70%, the mineral particles increased in number, and became more densely dispersed on cellulose substrates (Fig. 3d-2 and e-2). From the TEM images, it is hard to observe mineral crystals for sample M10 (Fig. 3a-3) and M30 (Fig. 3b-3). While a great number of mineral crystals (HA nanorods with size of less than 20 nm) can be observed when increasing the cellulose/HA mass ratios to 40***–***70% (Fig. 3c-3 to d-3), and all the mineral crystals are bound to the cellulose substrates, indicating a strong interaction between cellulose and the minerals. The morphology of the regenerated cellulose from NaOH/urea aqueous solution has also been investigated, and it was found that the regenerated cellulose was aggregate together to form big blocks with roughly surface and loose structure (Fig. S4). In the previous study of our group, the molecular weight of cellulose was measured to decrease from 34843–38894 (original cellulose) to 24300 (regenerated cellulose) after treated with NaOH/urea aqueous solution^45^, indicating that the cellulose was partially degraded under the strong alkaline condition. The coarse surface and degraded cellulose substrate make the as-prepared cellulose/HA nanocomposites more favorable for the application in biomedical fields, such as bone tissue engineering.

### Mineralization of cellulose/HA nanocomposites in SBF

**Figure 4:**
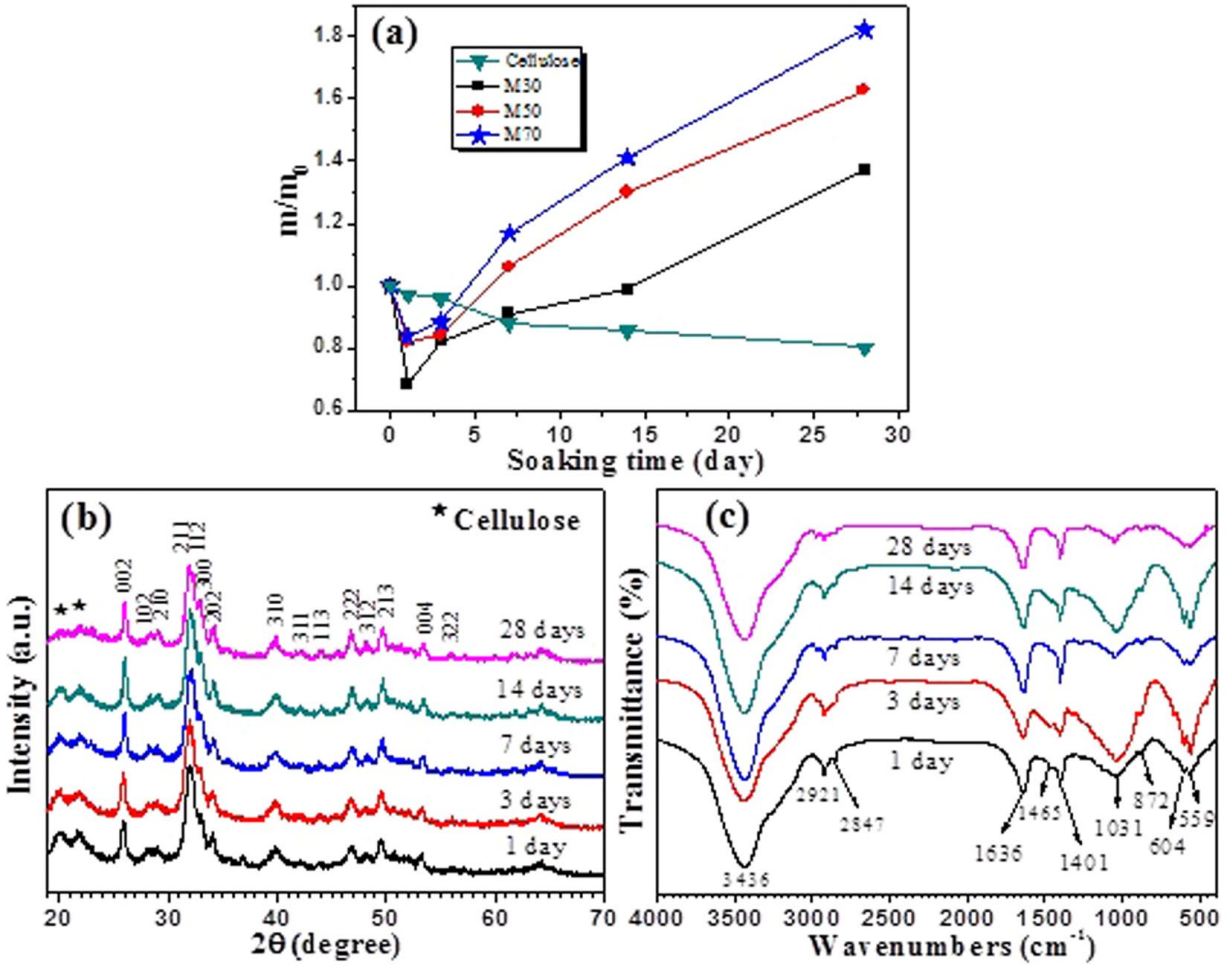
(**a**) Mass change of pure cellulose and the cellulose/HA nanocomposites (samples M30, M50, and M70) after soaking in SBF solution (pH 7.4) for different times. m/m_0_ is the sample mass at the respective time point divided by the initial mass; (**b**) XRD patterns and (**c**) FT-IR spectra of sample M70 after soaking in SBF solution (pH 7.4) for different times.

The *in vitro* behavior of the as-prepared cellulose/HA nanocomposites was studied to evaluate the biological response of the nanocomposites following immersion in simulated body fluid (SBF) for various periods (maximum of 28 days). Figure 4a shows the mass change of pure cellulose and the cellulose/HA nanocomposites (samples M30, M50, and M70) after soaking in SBF for different times. From which, one can see that the quality of pure cellulose gradually decreased with increasing the soaking time, and decreased to 0.82 times of the initial mass at 28 days. Maybe result from the slow degradation of cellulose in SBF since cellulose is essentially biodegradable^22^, or the mass loss during the refreshing of SBF solution because the SBF was reshed every day. The cellulose/HA nanocomposites prepared with different mass ratios of 30%, 50%, and 70% show the similar trend of mass change, which can be devided into two stages: the masses decreased in the first 1 day immersion in SBF, and subsequently increased in the masses with increase in the soaking duration. Such a mass change was also observed in previous study, which demonstrated that the formation of apatite on the surface of HA when soaking in SBF was attributable to the ion exchange between HA and the SBF solution^21^. The maximum masses were 1.37 (M30), 1.63 (M50), and 1.82 (M70) times of initial masses after 28 days of soaking, indicating that the nanocomposites prepared with high mass ratio of HA/cellulose possess high ability to induce the formation of HA crystals.

To investigate the transformation of cellulose/HA nanocomposites in SBF, the XRD and FT-IR measurements were performed on the samples after soaking in SBF for different times. From Fig. 4b, the peaks located around at 2*θ* = 20.2° and 21.90° are attributable to cellulose, and all the other peaks can be indexed to HA with hexagonal structure (JCPDS 09-0432). The intensities of the diffraction peaks of cellulose decreased when increase in soaking time, suggesting that the crystallinity of cellulose was decreased since cellulose is essentially biodegradable. In addition, the adsorption peak at 1669 cm^−1^ (carbonyl stretching in cellulose, Fig. 2B) disappeared after soaking in SBF (Fig. 4c), indicating that the cellulose substrates were partially degraded.

Previous literature proposed that the HA formation from metastable aqueous solution is usually preceded by a precursor phase, the amorphous calcium phosphate (Ca_x_H_y_(PO_4_)_z_·nH_2_O, n = 3~4.5, ACP) or octacalcium phosphate (Ca_8_H_2_(PO_4_)_6_, OCP), then the precursor calcium phosphate can hydrolyze into the more thermostable HA^44^. When OCP was the precursor, Ca^2+^ first complexes with another species before combining with PO_4_^3−^, which can ensure a more crystalline and ordered HA phase^46^. While if the precursor was ACP, the poorly crystalline mineral would be obtained, since ACP precipitation usually requires the rapid interaction between Ca^2+^ and PO_4_^3−^ at high supersaturation rather than precursor complexation with other species^47^. This can be responsible for the low crystallinity of HA in the sample after 28 days of soaking. The different precursors of these products obtained by soaking sample M70 in SBF for different times were also demonstrated by FT-IR analysis (Fig. 4c). The sharp peaks of PO_4_^3−^ (*v*_4_ bending mode of O–P–O) doublet at 604 and 559 cm^−1^ in the products obtained after 3 days and 14 days of soaking suggest that the precursor phase of HA was OCP in these two samples^46^. On the other hand, if the precursor was ACP, the peaks of PO_4_^3−^ (*v*_4_ bending mode of O–P–O) would be a broad singlet rather than a doublet^48^. Hence, the products obtained after 1 day, 7 days, and 28 days of soaking may be hydrolyzed from a mixture phases of OCP and ACP, since the intensities of the doublet peaks at 604 and 559 cm^−1^ were weakened in these samples. Although the apatite was hydrolyzed from different precursor phases, which was influenced by the conditions present in the solution during the precipitation reaction^46^, the as-prepared cellulose/HA nanocomposites exhibited good bioactivity, and with high ability to induce the formation of HA by soaking in SBF solution.

It is noted that the weak peaks at 1465 and 872 cm^−1^ originated from [CO_3_^2−^] can be observed in all of the products (Fig. 4c). Moreover, the Ca/P molar ratios were measured for 1.69, 1.63, 1.60, 1.55, and 1.54 corresponding to 1, 3, 7, 14, and 28 days of soaking, respectively, by ICP analysis. These results indicated that the HA crystals formed on the surface of the nanocomposites are carbonate-containing apatite, i.e. calcium-deficient carbonated hydroxyapatite (CdHA), which has been widely reported in previous literatures^21,25,44^. CdHA is more favourable for biomedical applications such as bone repair, since the naturally occurring calcium phosphate is usually carbonated and CdHA with a Ca/P molar ratio of less than 1.67^3^. On the other side, the CdHA shows a faster biodegradation rate than neat HA since the solubility of HA is lower than that of CdHA^2^, and the lower crystallinities of the CdHA crystals are also beneficial to the control of *in vivo* resorbability rates^49^.

**Figure 5:**
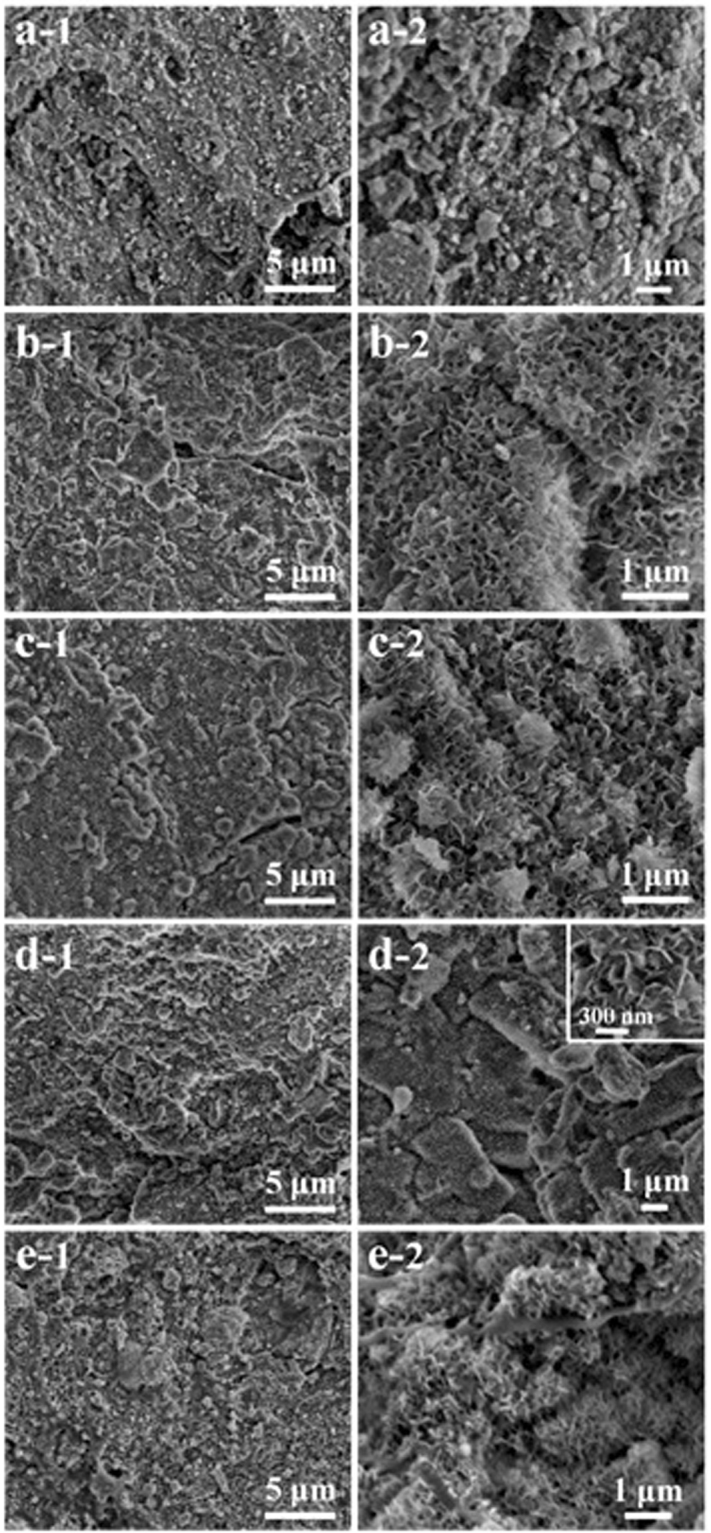
FE-SEM images of sample M70 after soaking in SBF solution (pH 7.4) for different times: (**a**) 1 day; (**b**) 3 days; (**c**) 7 days; (**d**) 14 days and (**e**,**f**) 28 days.

**Figure 6:**
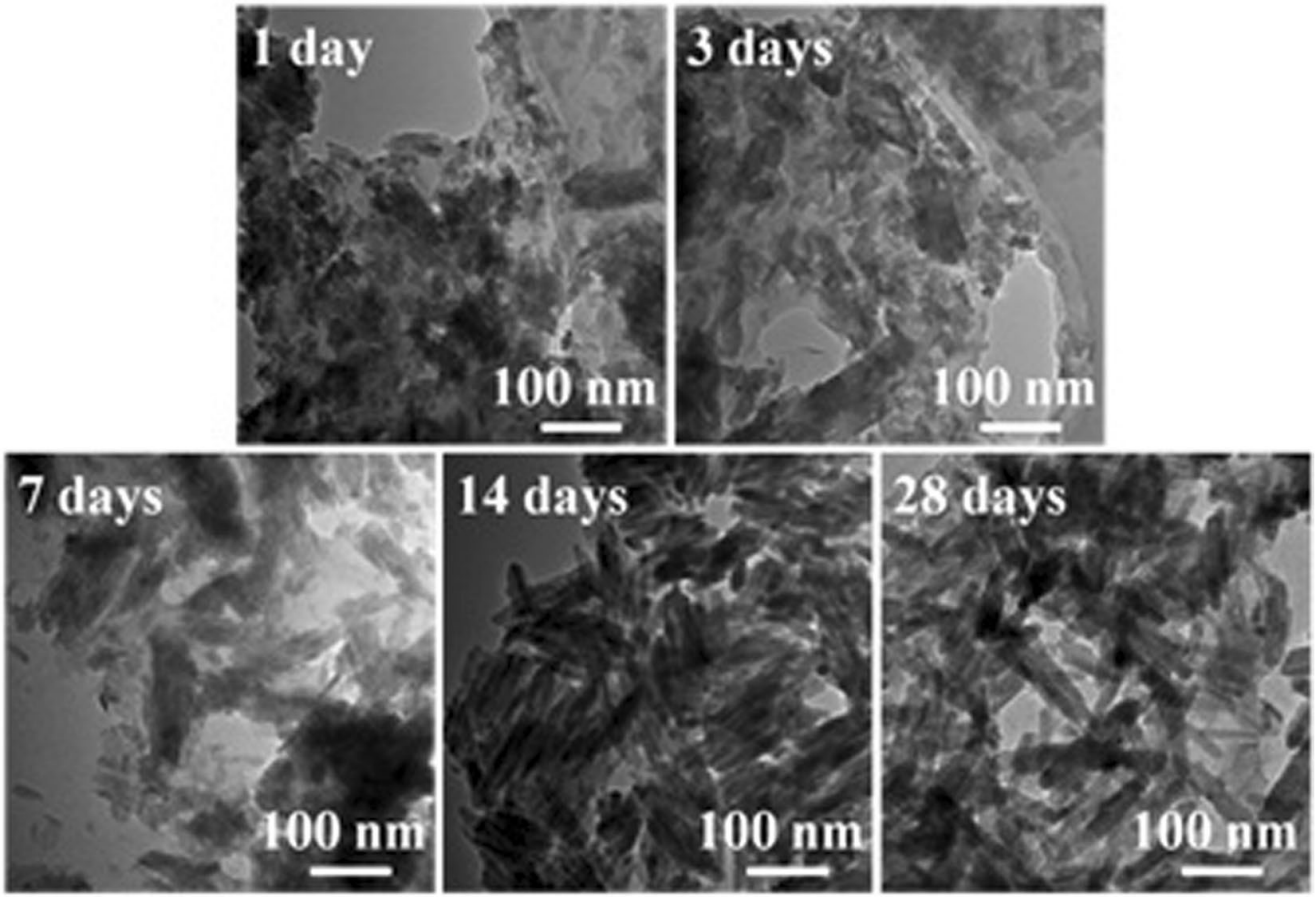
TEM images of sample M70 after soaking in SBF solution (pH 7.4) for different times.

Figure 5 shows the morphologies of sample M70 after different times of soaking. The HA particles in uneven size dispersed on cellulose substrates are observed after 1 day of soaking (Fig. 5a). After 3 days of soaking, numerous nanosheets have grown on the surface (Fig. 5b). The nucleation of these HA nanosheets prefers to occur in pores or chasms, where the size of the HA nanosheets is, obviously, much larger than that on the flat part of the surface. Increasing the soaking time to 7, 14, and 28 days, there is no significant change in the surface morphology, and the HA nanosheets can be observed in Fig. 5c–e, which are assembly of HA nanorods as observed by TEM images (Fig. 6). With increase in the soaking time, the quantity of these precipitates increase as well as the sizes of the HA nanorods. The pure cellulose after soaking in SBF for different times was also investigated by FE-SEM, as shown in Fig. S5. The cellulose with smooth surface can be observed by all of the soaking durations, indicating that the cellulose used in this study has weak ability to induce the formation of apatite.

### *In vitro* cytocompatibility tests and protein adsorption

As one of the ideal biomaterials, the synthetic HA has been investigated for applications in various biomedical fields owing to its excellent bioactivity and biocompatibility^8–10^. A key issue involved in these applications is the development of suitable HA materials with high porosity, suitable pore size, and highly interconnected pore structure^49^. In this study, the cellulose/HA nanocomposites had a specific surface area of 94.5 m^2^ g^−1^ and average pore size of 3.8 nm (Fig. S6). The relatively large specific surface area and nanoporous structure are favorable for application in protein adsorption since they can provide a large number of active sites and physical space for protein adsorption. Moreover, the zeta potential of the cellulose/HA nanocomposites was negatively charged in deionized water, while the Hb was positively charged (Fig. S7), demonstrating that the as-prepared cellulose/HA nanocomposites can adsorb Hb molecules through electrostatic attraction.

**Figure 7:**
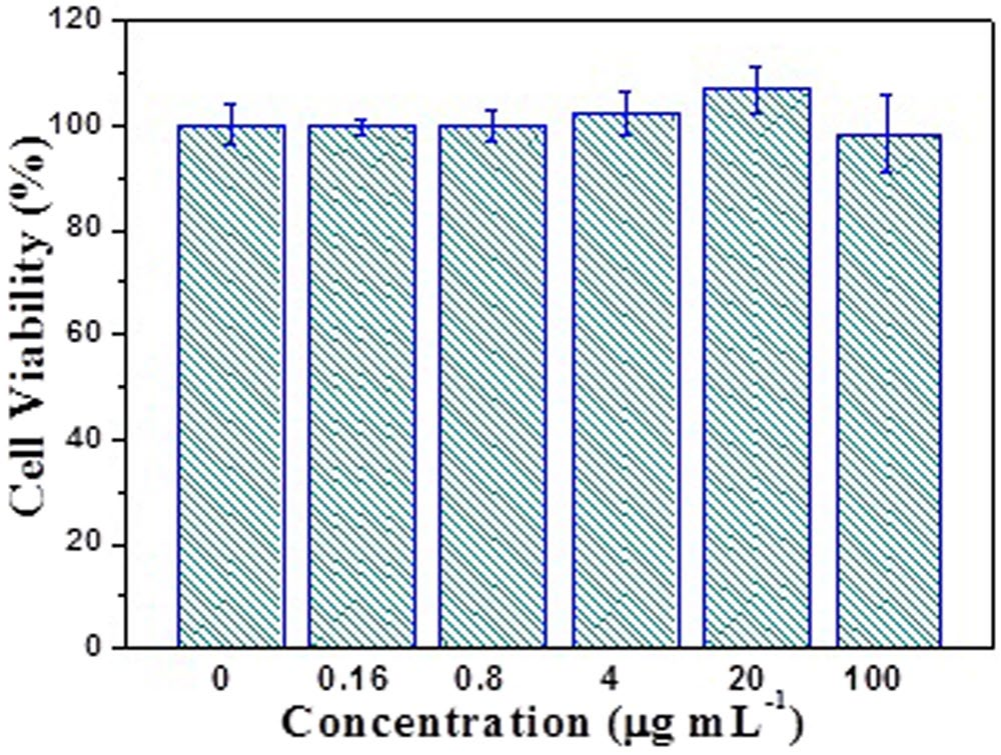
Cytotoxicity tests of the sample M70.

**Figure 8:**
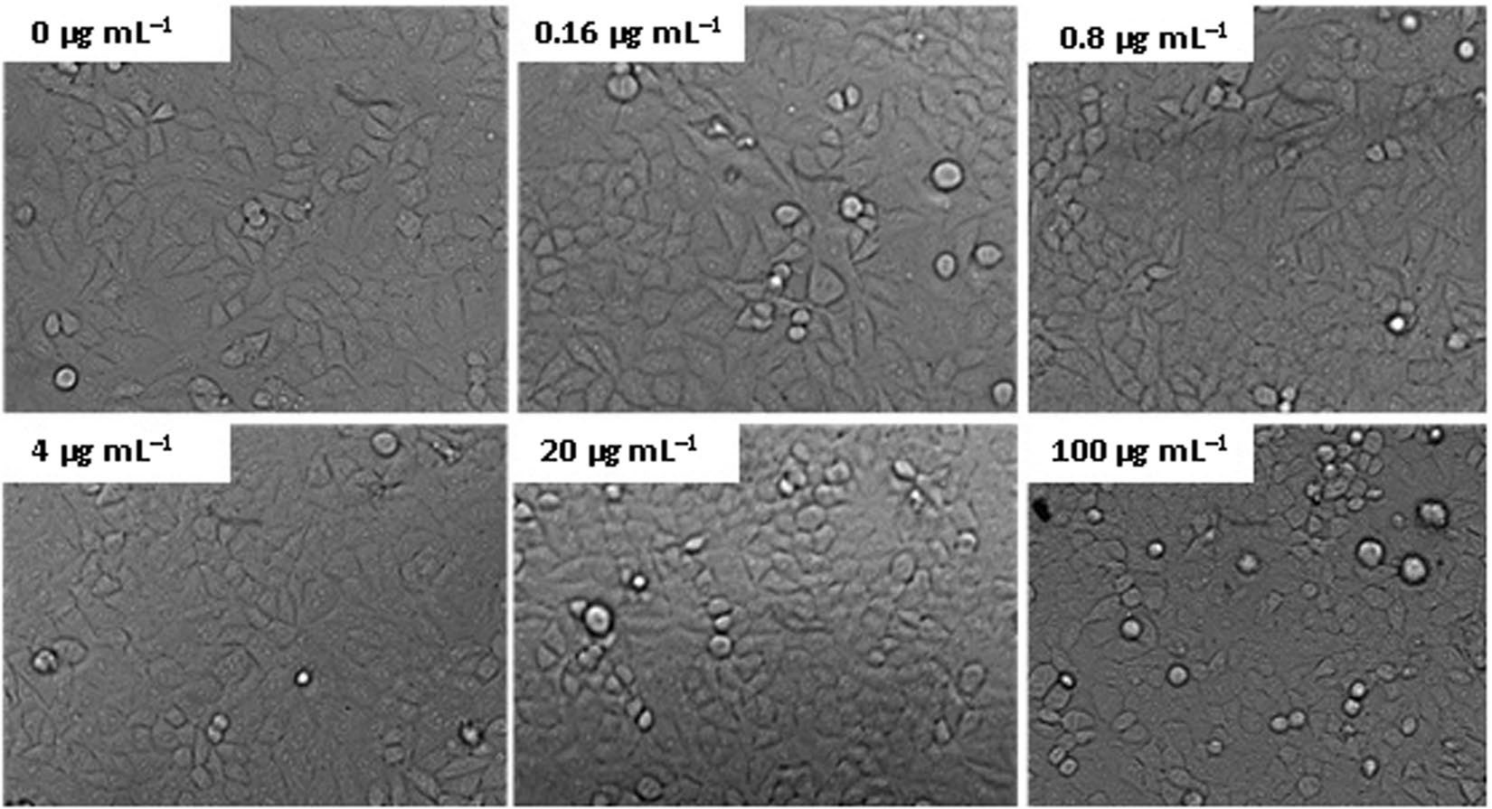
The optical images of human gastric carcinoma cells (MGC-803) treated with different concentrations of sample M70.

The cytotoxicity tests of cellulose/HA nanocomposites was also performed by MTT assay on human gastric carcinoma cells (MGC-803). As shown in Fig. 7, the MTT assay showed no notable toxicity since all of the cell viabilities were exceed 98% at the composites concentrations of 0–100 *μ*g mL^−1^, indicating that the as-prepared cellulose/HA nanocomposites have good cytocompatibility. Moreover, according to the optical images (Fig. 8), the MGC-803 cells could maintain a spindle morphology and a good physiological state after co-cultured with different concentrations of cellulose/HA nanocomposites. These results indicate that the as-prepared cellulose/HA nanocomposites have a high biocompatibility, which is consistent with the cell viability measured by MTT assay.

**Figure 9:**
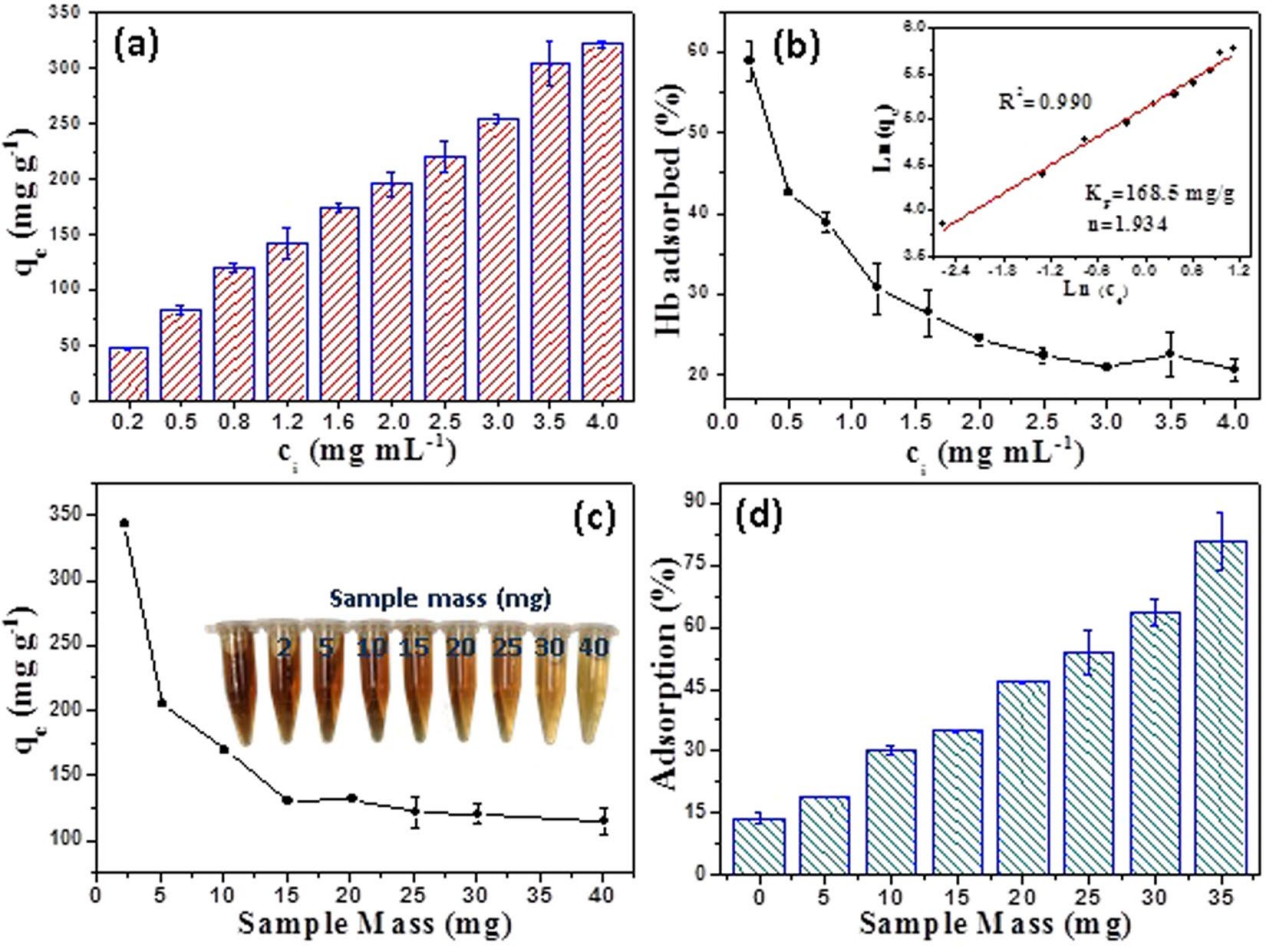
The Hb adsorption properties of the as-prepared cellulose/HA nanocomposites (sample M70): (**a**) the amount of Hb adsorbed at equilibrium (*q*_*e*_) as a function of initial concentrations (*c*_*i*_) of Hb; (**b**) Hb adsorption percentage as a function of *c*_*i*_ of Hb; the inset shows the exponential relationship of cellulose/HA nanocomposites between the amount of Hb adsorbed at *q*_*e*_ and equilibrium concentrations (*c*_*e*_) of Hb; (**c**) the amount of Hb adsorbed at *q*_*e*_ as a function of sample mass; the inset is the optical photographs of the Hb solutions (3 mg mL^−1^) before and after absorbed by the cellulose/HA nanocomposites with different sample masses (0–40 mg); (**d**) Hb adsorption percentage as a function of sample mass.

The protein adsorption properties of the as-prepared cellulose/HA nanocomposites (sample M70) are investigated using hemoglobin (Hb) as a model protein. The adsorption of Hb on the surface of cellulose/HA nanocomposites was supported by FT-IR analysis (Fig. S8). The peaks located at 1649 cm^−1^ and 1398 cm^−1^ (originated from Hb molecules) occurred after adsorption, indicating that the Hb molecules have been successfully adsorbed on the surface of cellulose/HA nanocomposites. The Hb adsorption properties of the as-prepared cellulose/HA nanocomposites were investigated at different initial Hb concentrations and different amounts of the sample. As shown in Fig. 9a, the amount of adsorbed Hb on the cellulose/HA nanocomposites increases with increasing initial concentration of Hb in the range of 0.2–4.0 mg mL^−1^ and reached 321.5 mg g^−1^ at a Hb initial concentration of 4.0 mg mL^−1^, which is higher than those reported for calcium phosphate carriers^9,29^. The cellulose/HA nanocomposites still did not saturated adsorption at a high Hb concentration of 4.0 mg mL^−1^, indicating that the cellulose/HA nanocomposites have a high protein adsorption capacity. The Hb adsorption percentage of the nanocomposites, the ratio of the amount of adsorbed Hb to the total mass of Hb in the solution, was decreased from 58.9% to 20.7% with increasing of Hb initial concentration (Fig. 9b). In addition, the amount of Hb adsorbed at equilibrium is consistent with the Freundlich adsorption model with a regression factor (R^2^) of 0.990 (the inset in Fig. 9b). The Freundlich isotherm (equation 1) represents the relationship between *q*_*e*_ and *c*_*e*_, where *q*_*e*_ (mg g^−1^) is the amount of Hb adsorbed at equilibrium, *c*_*e*_ (mg mL^−1^) is the equilibrium concentration of the solution, *K*_*F*_ and *n* are constants. According to the adsorption isotherm, the constants of *K*_*F*_ and *n* are 168.5 mg g^−1^ and 1.924, respectively. The value of *n* in the range of 1–10 suggesting a good adsorption^50^, thus the cellulose/HA nanocomposites are favorable for the adsorption of Hb.

1 $$\mathrm{Ln}{q}_{e}=\,\mathrm{Ln}{K}_{F}+\frac{1}{n}\,\mathrm{Ln}{c}_{e}$$

The Hb adsorption capacity of cellulose/HA nanocomposites with different sample masses at a Hb initial concentration of 3.0 mg mL^−1^ are shown in Fig. 9c and d. The amount of Hb adsorbed at equilibrium of the cellulose/HA nanocomposites was 343.2 mg g^−1^, and decreased sharply with increasing of sample mass from 2 mg to 40 mg (Fig. 9d), while the Hb adsorption percentage increases with increasing of sample mass, and reached a maximum of 80.9% (Fig. 9c). These results indicate that the as-prepared cellulose/HA nanocomposites have a relatively high protein adsorption capacity, which is also supported by the optical photographs of Hb aqueous solutions after adsorption (insets in Fig. 9d).

## Materials and Methods

Hemoglobin (Hb, molecular weight ~68000 Da) was purchased from Jinsui Biotech (Shanghai) Co., Ltd. The microcrystalline cellulose (molecular weight of 34,843–38,894, degree of polymerization (DP), DP = 215-240) was purchased from Sinopharm Chemical Reagent Co., Ltd. All other chemicals used in this study were purchased from Beijing Chemical Works, all of which were of analytical grades and used as received without further purification.

### Preparation of cellulose/HA nanocomposites

In a typical synthesis, for the preparation of cellulose solution, NaOH (2.8 g) and urea (4.8 g) were added into deionized water (32.4 mL) to form NaOH/urea aqueous solution. Then, the cellulose (1.0 g) was added into the above solution under magnetic stirring at room temperature. Afterwards, the solution was cooled to −12 °C for 12 h. For the synthesis of cellulose/HA nanocomposites, CaCl_2_ (0.111 g) and NaH_2_PO_4_·2H_2_O (0.093 g), at molar ratio Ca/P = 1.67, were added in the resulting cellulose solution, the mass ratio of HA/cellulose was set as 10% in the nanocomposites. Then the resulting suspension was subjected to sonication (Xin-Zhi, JY92-2D, Ti-horn, 20 kHz, 80 W/cm^2^) at ambient condition with a high-density ultrasonic probe immersed directly in the suspension. During the ultrasonic irradiation, the reaction system was cooled in an ice bath and the sonication was opened 2 s and closed 2 s for protecting the instrument. After 30 min, the product was separated by centrifugation, washed by deionized water for several times, and freeze-dried. Other samples were prepared by similar procedures but with varying experimental parameters, the details of the preparation conditions are shown in Table 1. The products with different mass ratios of HA/cellulose were used for further assessment, and the cellulose/HA nanocomposites with HA/cellulose mass ratios of 10, 30, 40, 50, 70 wt% were labeled as M10, M30, M40, M50, and M70, respectively.

### Characterizations

X-ray powder diffraction (XRD) patterns of the as-prepared cellulose/HA nanocomposites were recorded in 2*θ* range from 10° to 70° on a Rigaku D/Max 2200-PC (Tokyo, Japan), operating with Cu Kα radiation (λ = 0.15418 nm) and graphite monochromator. Fourier-transform infrared (FT-IR) spectroscopic measurements were carried out on Bruker VERTEX 70 V (Karlsruhe, Germany) with the wavenumber range from 4000 to 400 cm^−1^ at 0.4 cm^−1^ resolution and 64 scans per sample. Deconvolution of the cellulose OH bands (3600–3100 cm^−1^) was performed by fitting the experimental band to Gaussian-Lorentzian components using PeakFitv.4.12 software (SeaSolve Software: Framingham, MA, USA). Field emission scanning electron microscopy (FE-SEM) were observed with Hitachi SU8010 (Tokyo, Japan). All samples were Au coated prior to examination by FE-SEM. The sizes of HA crystals in the nanocomposites were obtained from the FE-SEM image using Image J software (NIH, Bethesda, Maryland, USA). Transmission electron microscopy (TEM) images were performed on Hitachi HT-7700 (Tokyo, Japan). The Brunauer-Emmett-Teller (BET) specific surface area and pore-size distribution were measured using Quantachrome Autosorb-iQ2-MP (Florida, USA). The Ca/P molar ratio of the product was calculated from the Ca and P elemental contents in the sample, which were obtained using an inductively coupled plasma (ICP) optical emission spectrometer, Horiba JY2000-2 (Paris, France). The Hemoglobin (Hb) concentrations were analyzed by a ultraviolet-visible spectrophotometer Techcomp UV2310 II (Shanghai, China) at wavelength of 405 nm.

### The transformation of cellulose/HA nanocomposites in simulated body fluid

The powdered sample (0.05 g) was soaked in 50 mL simulated body fluid (SBF, pH = 7.4) at 37 °C for 1, 3, 7, 14, and 28 days, the SBF solution was refreshed every day. After that, the samples were separated by centrifugation, freeze-dried, and weighed for further characterization. The SBF was prepared by the method reported by Kokubo and coworkers^44^. As shown in Table S1, a certain quality of NaCl, NaHCO_3_, KCl, K_2_HPO_4_·3H_2_O, MgCl_2_·6H_2_O, 1.0 M HCl, CaCl_2_, Na_2_SO_4_, and tris(hydroxymethyl) aminomethane ((CH_2_OH)_3_CNH_2_) were added one by one after each reagent was completely dissolved 800 mL deionized water, and 1.0 M HCl solution (0–5 mL) was used for adjusing pH at last. The pH of the solution was buffered at 7.4 ± 0.01 (36.5 ± 0.5 °C) by (CH_2_OH)_3_CNH_2_ and HCl solution. After the solution was natural cooled to 20 °C, the total volume of the solution was adjusted to 1000 mL by adding deionized water. The SBF obtained could be stored at 5 °C for a month without degradation.

### *In vitro* cell cytotoxicity tests

The cell cytotoxicity test of the as-prepared cellulose/HA nanocomposites was carried out on human gastric carcinoma cells (MGC-803). The cells were cultured in RPMI-1640 medium supplemented with 10% fetal bovine serum and 1% penicillin-streptomycin at 37 °C for 48 h. Then, the cells were seeded in 96-well flat-bottom microassay plates at a concentration of 1 × 10^4^ cells per milliliter and cultured for 24 h. The sterilized powdered sample was added into the wells at concentrations ranging from 0.16–100 *μ*g mL^−1^ and co-cultured with the cells for 48 h. The sample-free tissue culture plate was used as control. The cell viability was quantified by 3-(4,5 dimethylthiazol-2-yl)-2,5-diphenyltetrazollium bromide (MTT) assay, and the data represents the mean value of three parallel measurements. Cell images of MGC-803 cells co-cultured with different concentrations of cellulose/HA nanocomposites for 48 h were obtained using an Olympus GX71 fluorescence microscope.

### *In vitro* protein adsorption

Hemoglobin was chosen as a model protein for the investigation. The protein adsorption experiments at different protein concentrations were performed as follows: the powdered samples (5 mg) were immersed in aqueous solutions that contained various concentrations of Hb (2 mL, 0–4.0 mg mL^−1^). After ultrasonic treatment for 1 min, each solution was shaken in a double-layer shaking incubators ZWYR-2102C at a constant rate at 37 °C for 6 h. Then the solution was centrifuged, and the amount of Hb in the supernatant was measured by UV-vis absorption at a wavelength of 405 nm. The sample shaked with Hb aqueous solution (3.0 mg mL^−1^) was centrifuged, washed with deionized water and freezed dried, and further characterized by FT-IR. The protein adsorption experiments with different sample masses were also performed: the powdered cellulose/HA nanocomposites (0–40 mg) were immersed in aqueous solutions containing Hb concentration of 3.0 mg mL^−1^ and each solution was shaken at a constant rate at 37 °C for 6 h. Then the solution was centrifuged, and the amount of protein in the supernatant was measured by UV-vis absorption at a wavelength of 405 nm.

## Conclusions

In summary, this paper describes a facile and green route for the synthesis of cellulose/HA nanocomposites by the sonochemical method in NaOH/urea aqueous solution. The *in vitro* behavior of the as-prepared cellulose/HA nanocomposites was studied to evaluate the biological response of the nanocomposites following immersion in SBF for various periods (maximum of 28 days). The HA formed on the surface of the nanocomposites was carbonate-containing, which is more favourable for biomedical applications. The calcium phosphate nanosheets (assembly of HA nanorods) were mineralized on the surface of the nanocomposites, and maximum mass of the nanocomposites was reached 1.82 times of initial mass after 28 days of soaking. Moreover, the as-prepared cellulose/HA nanocomposites have good cytocompatibility, and show a relatively high protein adsorption ability (321.5 mg g^−1^) using hemoglobin as a model protein. These results indicate that the cellulose/HA nanocomposites are promising for applications in various biomedical fields such as tissue engineering and protein/drug delivery.

## Electronic supplementary material

Supplementary information
