# Supplementary material for: Sonochemical synthesis of cellulose/hydroxyapatite nanocomposites and their application in protein adsorption
Source: Sci Rep. 2018 May 29;8:8292. doi: 10.1038/s41598-018-25566-7 (PMC5974341; doi:10.1038/s41598-018-25566-7)
Supplement: Supplementary file 1 — Supplementary information [file 41598_2018_25566_MOESM1_ESM.docx]

Support information

Sonochemical synthesis of cellulose/hydroxyapatite nanocomposites and their application in protein adsorption

Lian-Hua Fu,^1,2,3^ Chao Qi,^2^ Yan-Jun Liu,^1^ Wen-Tao Cao,^1^ and Ming-Guo Ma^1,^^[[1]](#footnote-1)^*

*^1^Beijing Key Laboratory of Lignocellulosic Chemistry, College of Materials Science and Technology, Beijing Forestry University, Beijing 100083, PR China*

*^2^Guangdong Key Laboratory for Biomedical Measurements and Ultrasound Imaging, Laboratory of Evolutionary Theranostics, School of Biomedical Engineering, Health Science Center, Shenzhen University, Shenzhen 518060, China*

*^3^Key Laboratory of Optoelectronic Devices and Systems of Ministry of Education and Guangdong Province, College of Optoelectronic Engineering, Shenzhen University, Shenzhen 518060, China*

**Table S1.** The reagents used for the preparation of SBF.

| Order | Reagent | Amount |
| --- | --- | --- |
| 1 | NaCl | 8.035 g |
| 2 | NaHCO_3_ | 0.355 g |
| 3 | KCl | 0.225 g |
| 4 | K_2_HPO_4_·3H_2_O | 0.231 g |
| 5 | MgCl_2_·6H_2_O | 0.311 g |
| 6 | 1.0 mol/L HCl | 39 mL |
| 7 | CaCl_2_ | 0.292 g |
| 8 | Na_2_SO_4_ | 0.072 g |
| 9 | (CH_2_OH)_3_CNH_2_ | 6.118 g |
| 10 | 1.0 mol/L HCl | 0-5 mL |


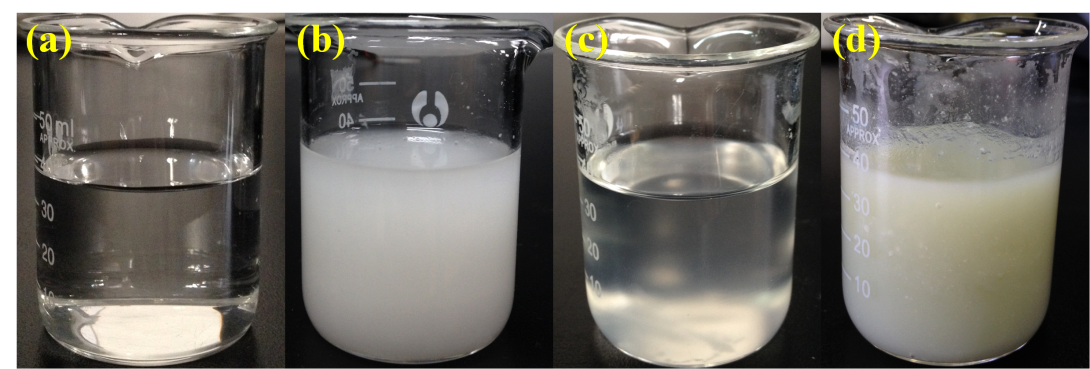


**Figure S1.** Photographs of (a) NaOH/urea aqueous solution, (b) cellulose dispersed in NaOH/urea aqueous solution, (c) after cooled to -12 °C for 12 h, and (d) after treated with ultrasonic irradiation for 30 min (sample M70).

**Figure S2.** Deconvoluted FT-IR spectra of pure cellulose (top) and cellulose/HA nanocomposites (sample M70, bottom).


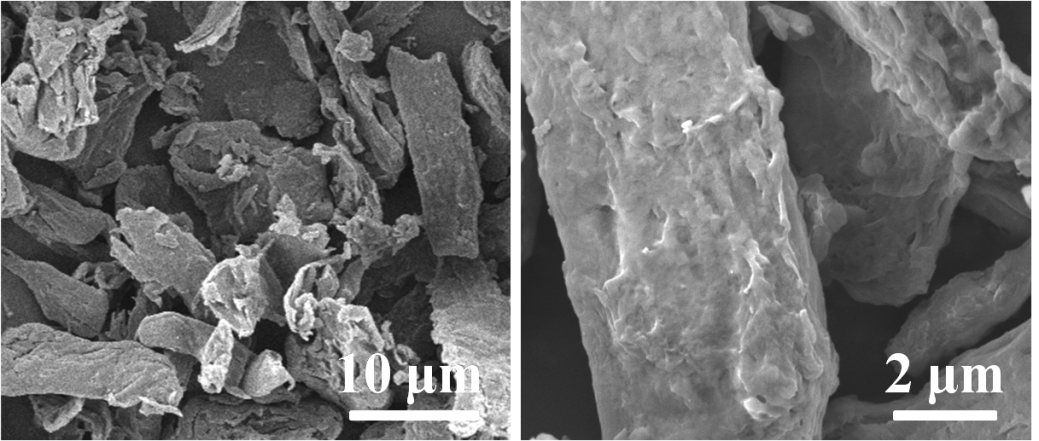


**Figure S3.** (a) FE-SEM images of the original cellulose.


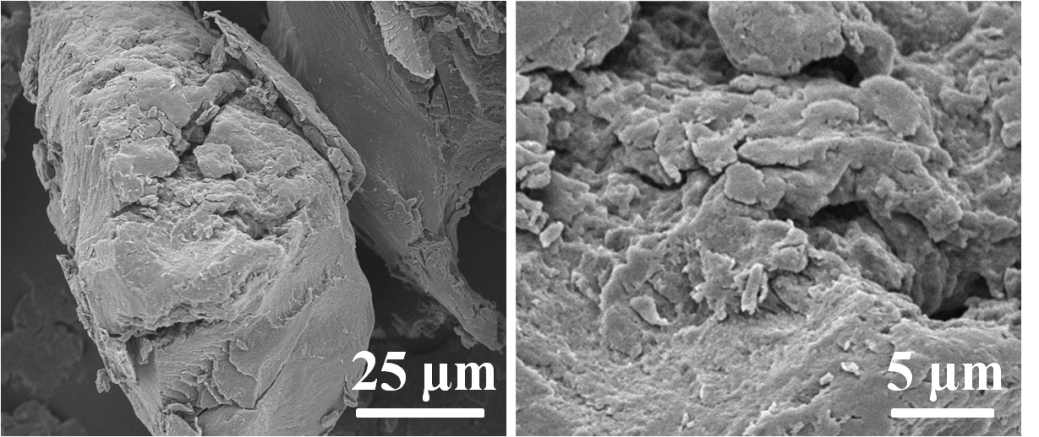


**Figure S4.** (a) FE-SEM images of cellulose regenerated from NaOH/urea aqueous solution.


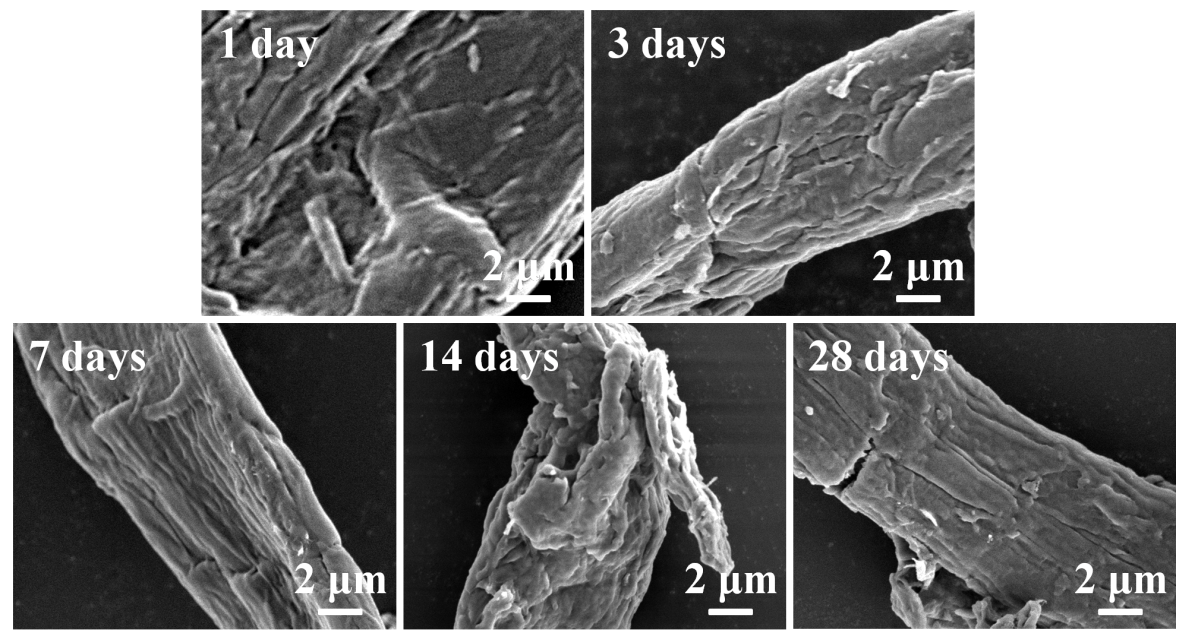


**Figure S5.** SEM images of cellulose after soaking in SBF solution (pH 7.4) for different times.

**Figure S6.** N_2_ adsorption-desorption isotherm (a) and BJH desorption pore-size distribution curve (b) of sample M70.

**Figure S7.** Zeta potentials of pure Hb and sample M70 in deionized water.

**Figure S8.** FT-IR spectra of pure Hb, and the sample M70 before and after Hb adsorption.

1. * Corresponding author. Tel.: +86-10-62337250; Fax.: +86-10-62336903.

   E-mail address: mg_ma@bjfu.edu.cn (M.-G. Ma). [↑](#footnote-ref-1)
